# Mechanical mechanism study of upper airway collapse and twin block treatment in a patient with mandibular retrognathia using fluid-structure interaction simulation

**DOI:** 10.1186/s12903-025-07252-z

**Published:** 2025-12-05

**Authors:** Jingying Wang, Yu Han, Shuai Chen, Mingrui Li, Chunhian Lee

**Affiliations:** 1 School of Nuclear Science, Energy and Power Engineering, Shandong University, Jinan, 250061 China; 2 Department of Stomatology, Jinan Central Hospital, Shandong First Medical University & Shandong Academy of Medical Sciences, Jinan, 250013 China

**Keywords:** Mandibular retrognathia, Upper airway collapse, Twin block, Fluid-structure interaction (FSI) simulation

## Abstract

**Background:**

The purpose of this study was to investigate the mechanical mechanism of upper airway collapse and orthodontic treatment in a child with mandibular retrognathia.

**Methods:**

Fluid-structure interaction (FSI) simulation was used to evaluate the collapse mechanism of upper airway and orthodontic mechanism of Twin Block (TB) in a patient with mandibular retrognathia. The upper airway model was 3D printed by lithography technology, and the FSI boundary conditions of airway soft tissue collapse and biomechanical parameters of oropharynx were obtained by in vitro experiments.

**Results:**

The results showed that the maximum negative pressure of oropharynx before treatment was located in the posterior wall, and the pressure gradient was larger than that of other parts. After treatment, the maximum negative pressure was limited to a small area of the anterior oropharynx wall, which decreased from − 2741.81 Pa to -1767.54 Pa, and the pressure gradient also decreased significantly. And the maximum deformation was reduced from 3.44 mm to less than 1 mm, which was reduced by more than 70%. Pearson correlation test showed that the change rate of the cross section area (α) was positively correlated with pressure drop (*P* < 0.05), and the closer to 1 the α value reached, the smaller the oropharynx pressure drop was. The larger the aspect ratio was, the smaller the maximum negative pressure was (*P* < 0.05).

**Conclusions:**

The collapse site of the upper airway in the presented child with mandibular retrognathia was not necessarily consistent with the narrowest part of the upper airway, and the cross-section shape and minimum pressure of the airway played a crucial role in affecting the collapse of the upper airway.

## Introduction

Mandibular retrognathia is one of the common craniofacial deformities in Chinese children [1]. Its main feature is that the mandible is in a relatively backward position to the maxilla, resulting in backward displacement of the tongue and hyoid bone leading to upper airway stenosis, thus affecting the patients’ ventilation function and normal craniofacial growth and development [2]. In severe cases, obstructive sleep apnea-hypopnea syndrome (OSAHS) can be induced. Previous studies mainly focused on pathophysiology to explore the pathogenesis of OSAHS [3, 4]. In recent years, more and more scholars have paid attention to the mechanical pathogenesis of OSAHS. The current known mechanical mechanism is mainly studied by establishing Starling resistance model to calculate the critical pressure of the upper airway and establishing biomechanical model to simulate the mechanical properties of the soft tissue of the upper airway [5, 6]. The upper airway collapse is defined as ≥ 75% obstruction from the morphological aspect [7]. In terms of critical pressure, it is defined as the minimum intraluminal pressure that keeps the upper airway open to the surrounding tissues. Positive airway pressure therapy is considered the gold standard treatment for OSAHS, but the success rate is only 50% [8]. A key unresolved issue is the factors that affect treatment outcomes and whether it is feasible to predict treatment responses before treatment is implemented. Computational fluid dynamics (CFD) simulation can help doctors better understand the biomechanics of upper airway collapse [9], thereby contributing to the development of new orthopedic techniques to prevent airway collapse more effectively [10].

At present, CFD and FSI simulations are used as important means to study airflow dynamics characteristics of the upper airway [11]. The latter can quantify the airway deformation characteristics by simulating the interaction between the airflow and the structures around the upper airway, and the response of the soft tissue when interacting with the airflow depends on the material properties and mechanical models applied during the FSI simulation [12]. Recently, a number of FSI studies have reported the aerodynamic characteristics of OSAHS patients. However, average elastic modulus and Poisson’s ratio in previous literatures are often used [13–15], resulting in the mismatch between respiratory flow boundary conditions and structural mechanical parameters and models around the upper airway. Therefore, it is difficult to accurately quantify the degree and mode of upper airway deformation under corresponding mechanical conditions. On the other hand, the mechanism leading to collapse is still unclear. Some studies believe that upper airway collapse occurs during inspiratory period [15, 16], while others find that the upper airway area during expiratory period is smaller [17, 18], which makes it more prone to collapse. In addition, with few exceptions [10], most previous studies lack experimental verification of FSI methodology.

Our basic hypothesis is that anatomic abnormalities leading to airway stenosis may increase the risk and severity of OSAHS by generating higher negative pressure within the upper airway. At the same time, TB is an effective means of correcting mandibular retrognathia [19–23]. Therefore, this study aims to obtain the boundary conditions of upper airway collapse through in vitro model experiments, and then study the pathogenesis of OSAHS and the treatment mechanism of TB from the perspective of mechanics by means of FSI simulation, so as to explore the risk factors leading to upper airway collapse.

## Materials and methods

### Research object

In this study, a growth and development patient of Class II, Division 1 with mandibular retrognathia was selected, the age was 10.0 years old, and the body mass index was 18.5 kg/m^2^. This study was approved by the ethics committee of jinan central hospital (NO.SZR2024-001) with informed consent of patients and the guardians.

### Methods

#### CBCT image acquisition

The same operator scanned patients while awake using the New Tom 5G CBCT. Voxel resolution was 0.3 mm, and scanning parameters were set at 110 kv and 5 mA. The patient was in a supine position during CBCT image acquisition, with the Frankfort plane perpendicular to the ground, maintaining intercuspal occlusion. The CBCT data obtained were output and stored in Digital imaging and communications in medicine (DICOM) format.

#### 3D upper airway model segmentation and reconstruction

Data in DICOM format was imported into Mimics 10.0 software (Materialise NV, Leuven, Belgium). The upper airway and surrounding soft and hard tissues were separated based on the threshold values of −1024 ~ −480 Hounsfield units (HU), and discrete pixels were removed using the region growing tool. The upper airway was divided into four parts: nasal cavity, nasopharynx, oropharynx and hypopharynx by identifying the posterior nostril, the level of the soft palate and the upper margin of the epiglottis in the median sagittal plane. Finally, the upper airway 3D model was exported in the format of stereolithography (STL).

#### In vitro experiment of collapsible upper airway

**Fig. 1:**
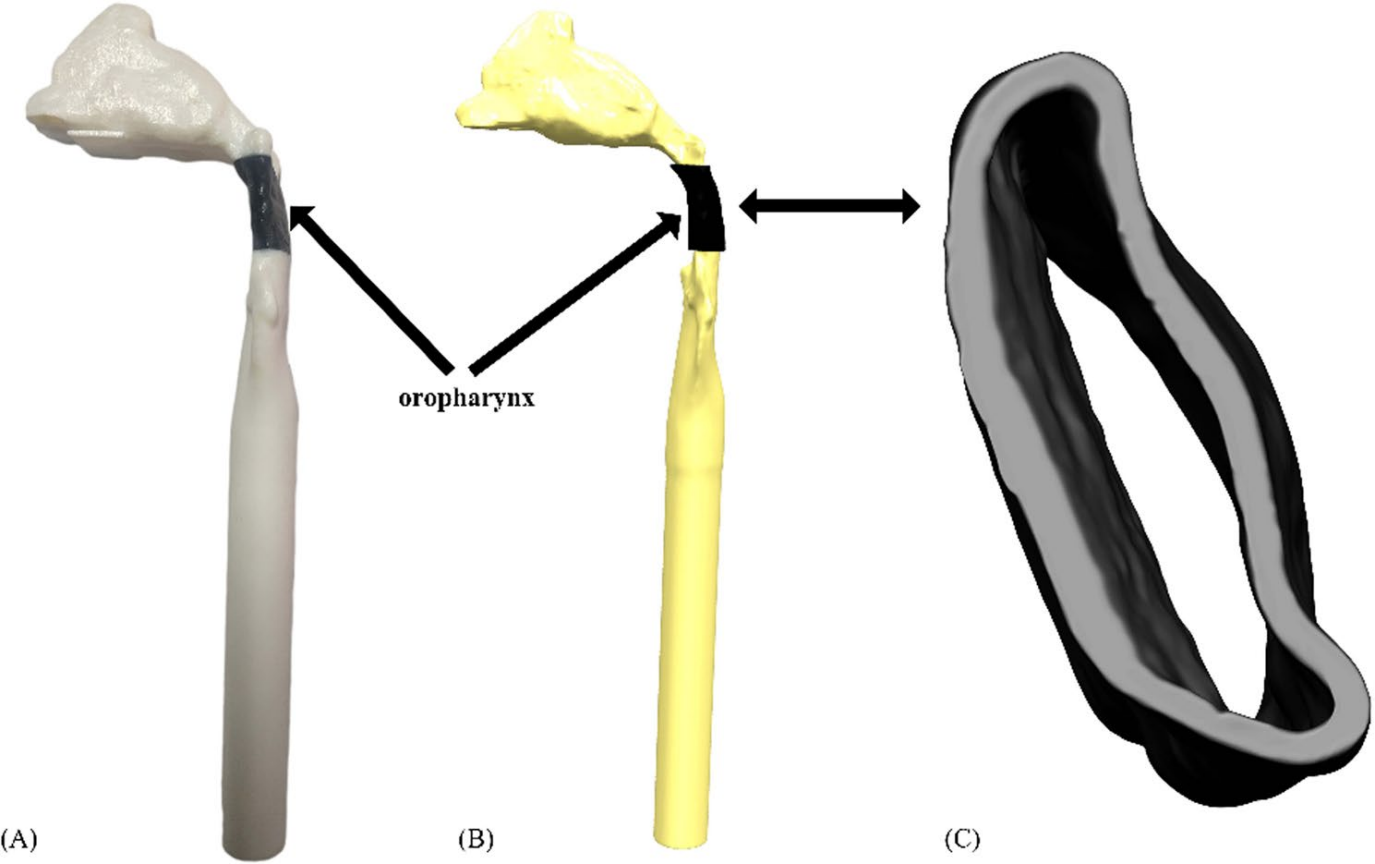
FSI model of human upper airway. (**A**) 3D printing integrated upper airway model; (**B**) 1:1 scale FSI model; (**C**) oropharynx soft tissue with a wall thickness of 2 mm

A 1:1 continuous upper airway model with a wall thickness of 2 mm was made using a combination of 3D printing and soft rubber molding (Fig. 1). The rigid part is photosensitive resin, and the foldable part, namely the oropharynx, is made of soft adhesive material. The elastic modulus of oropharynx soft rubber was 500 kPa, Poisson’s ratio was 0.495, and the density was 1800 kg/m^3^.

 In vitro upper airway collapse experiments were conducted using the experimental equipment from a previous study [24] by gradually increasing the flow of the air supply device until the oropharynx soft rubber collapsed. Since it was difficult to reach the pressure required for upper airway collapse when the nasal cavity was open on both sides, the right nostril was blocked to reduce the oropharyngeal pressure. Finally, obvious oropharynx deformation was achieved at a velocity of 6.3 m/s. At the same time, a precision displacement platform (precision 0.05%) was used to record the displacement at the maximum deformation of the soft rubber under stable flow, so as to facilitate the validation of subsequent FSI simulation.

#### Two-way FSI simulation

##### Upper airway meshing

**Fig. 2:**
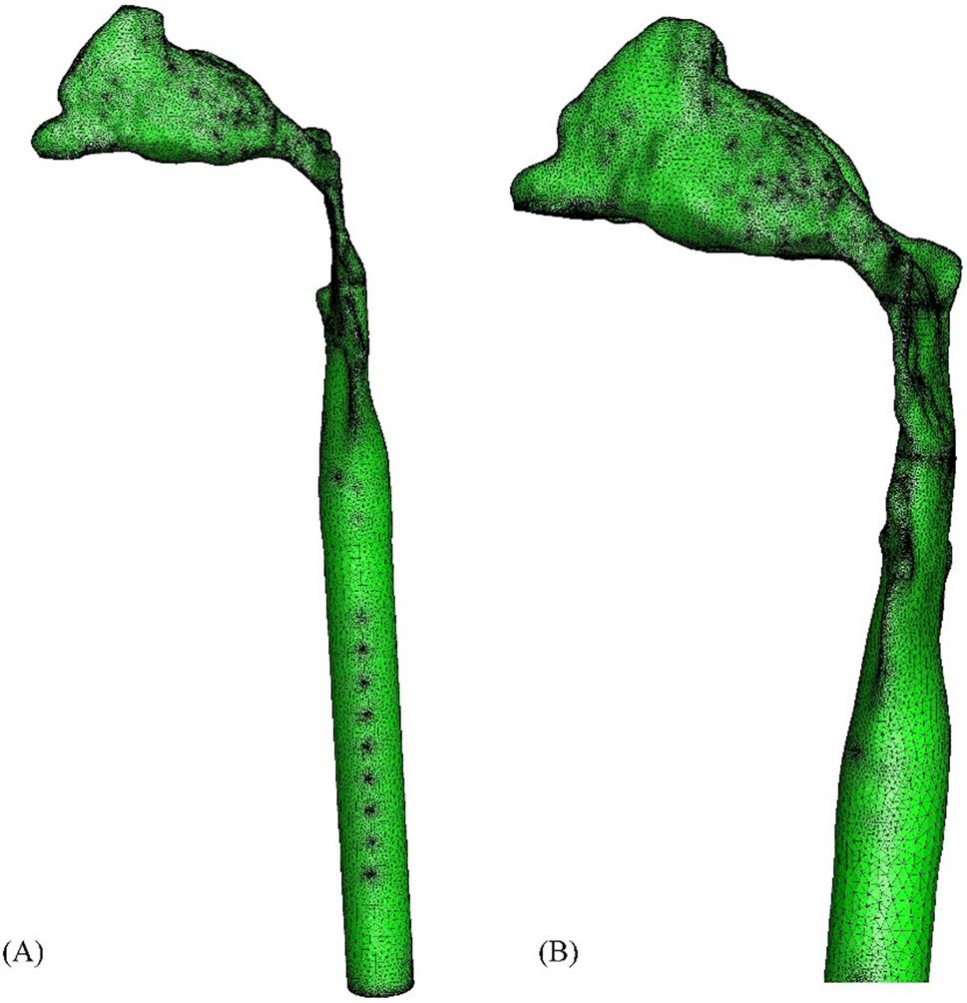
Grid of upper airway. (**A**) Overall image; (**B**) Local enlarged image

The upper airway in STL format was converted to iges format by reverse reconstruction and imported into Ansys workbench 19.2 (ANSYS, Inc, Canonsburg, USA) software. Fluent-meshing was used to generate mesh for the upper airway, and 5 maximum inflation layers were set on average, with a transition ratio of 0.272 and a growth rate of 1.2%. A tetrahedral volume grid with a maximum skew of 0.84 was generated (Fig. 2).

##### Boundary conditions for upper airway fluid domain

1. Consistent with the in vitro experiment, wall boundary conditions were applied in the right nostril to simulate the condition of right nasal obstruction, and the left nostril was set as an atmospheric pressure outlet.
2. The lower end of the pharynx was set as the velocity inlet, the tidal volume was edited as the standard sine function according to the data measured in vitro, and the time to reach the peak inspiratory value was set as 1.0 s.
3. Smoothing and remeshing were used to create the dynamic mesh region, and the diffusion coefficient was set to two to maintain the quality of the deformed mesh.
4. As a non-slip wall, the upper airway wall was simulated using standard k-ω, which has been validated in previous study [24].

##### Boundary conditions for oropharynx structure domain

1. Define the properties of oropharynx soft tissue based on mechanical test data.
2. Fix the upper and lower boundaries of the oropharynx, that is, the upper and lower junction area between the oropharynx and the upper airway.
3. A FSI interface was created, and the inner wall of the oropharynx was set as a Fluid Solid Interface.
4. FSI simulation was carried out within 0–1 s, the time step was set to 0.001 s, and the number of iterations was set to 80.

### Measuring items

1. Fluid domain related parameters: a contour was established every 1 mm along the Z axis of the upper airway to calculate the maximum negative pressure, maximum flow rate and maximum oropharynx resistance of the upper airway before and after treatment.
2. Solid domain related parameters: maximum upper airway deformation at each breathing moment, and the compliance of the oropharynx tissue (the area of the maximum deformation of the cross section and the slope of the pressure curve) was calculated, where the pressure was the area-weighted average pressure. The aspect ratio (AR) corresponding to the plane with the largest change in the cross section area of oropharynx soft tissue is defined as the ratio of the maximum anteroposterior size to the maximum transverse size. The change rate of oropharynx cross section (α) [25] is defined as follows: α_1_ and α_2_ represent the corresponding values of 0.4 s, 0.6 s, 0.8 s and 1.0 s before treatment, and α_3_ and α_4_ correspond to α_1_ and α_2_ respectively, representing the corresponding values of each breathing moment after treatment.
  1 $$\alpha_1=\frac{\mathrm{top}\;\mathrm{cross}\;\mathrm{section}\;\mathrm{area}\;\mathrm{of}\;\mathrm{oropharynx}}{\mathrm{the}\;\mathrm{area}\;\mathrm{where}\;\mathrm{the}\;\mathrm{oropharynx}\;\mathrm{cross}\;\mathrm{section}\;\mathrm{changes}\;\mathrm{the}\;\mathrm{most}}$$

2 $$\alpha_2=\frac{\mathrm{the}\;\mathrm{area}\;\mathrm{where}\;\mathrm{the}\;\mathrm{oropharynx}\;\mathrm{cross}\;\mathrm{section}\;\mathrm{changes}\;\mathrm{the}\;\mathrm{most}}{\mathrm{the}\;\mathrm{lowest}\;\mathrm{cross}\;\mathrm{section}\;\mathrm{area}\;\mathrm{of}\;\mathrm{the}\;\mathrm{oropharynx}}$$

## Results

### Fluid domain analysis

#### Upper airway flow velocity

**Fig. 3:**
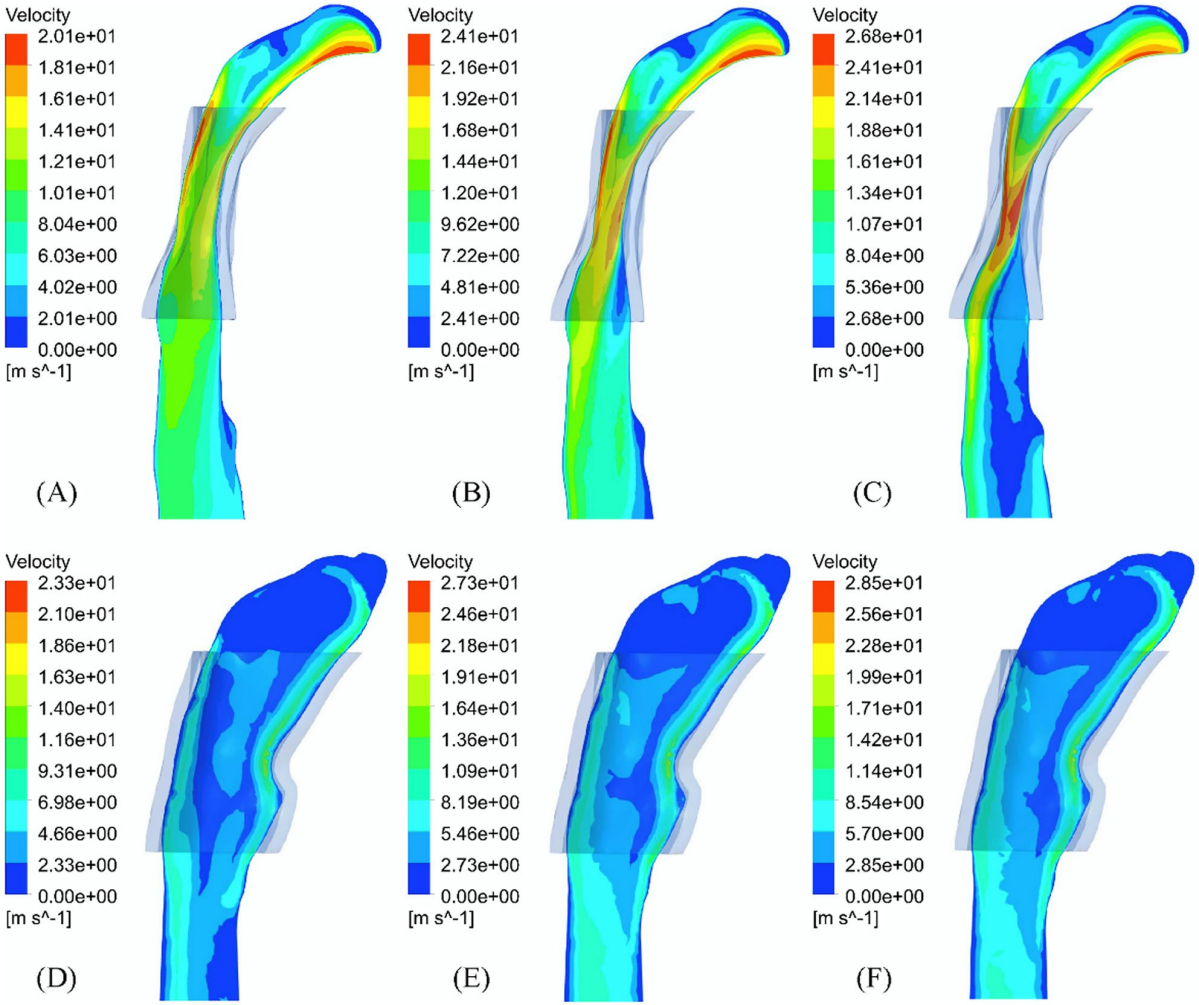
Velocity contour map of upper airway profile (transparent shadow is initial boundary of oropharynx): (**A**) 0.6 s, (**B**) 0.8 s and (**C**) 1.0 s before treatment; (**D**) 0.6 s, (**E**) 0.8 s and (**F**) 1.0 s after treatment

**Fig. 4:**
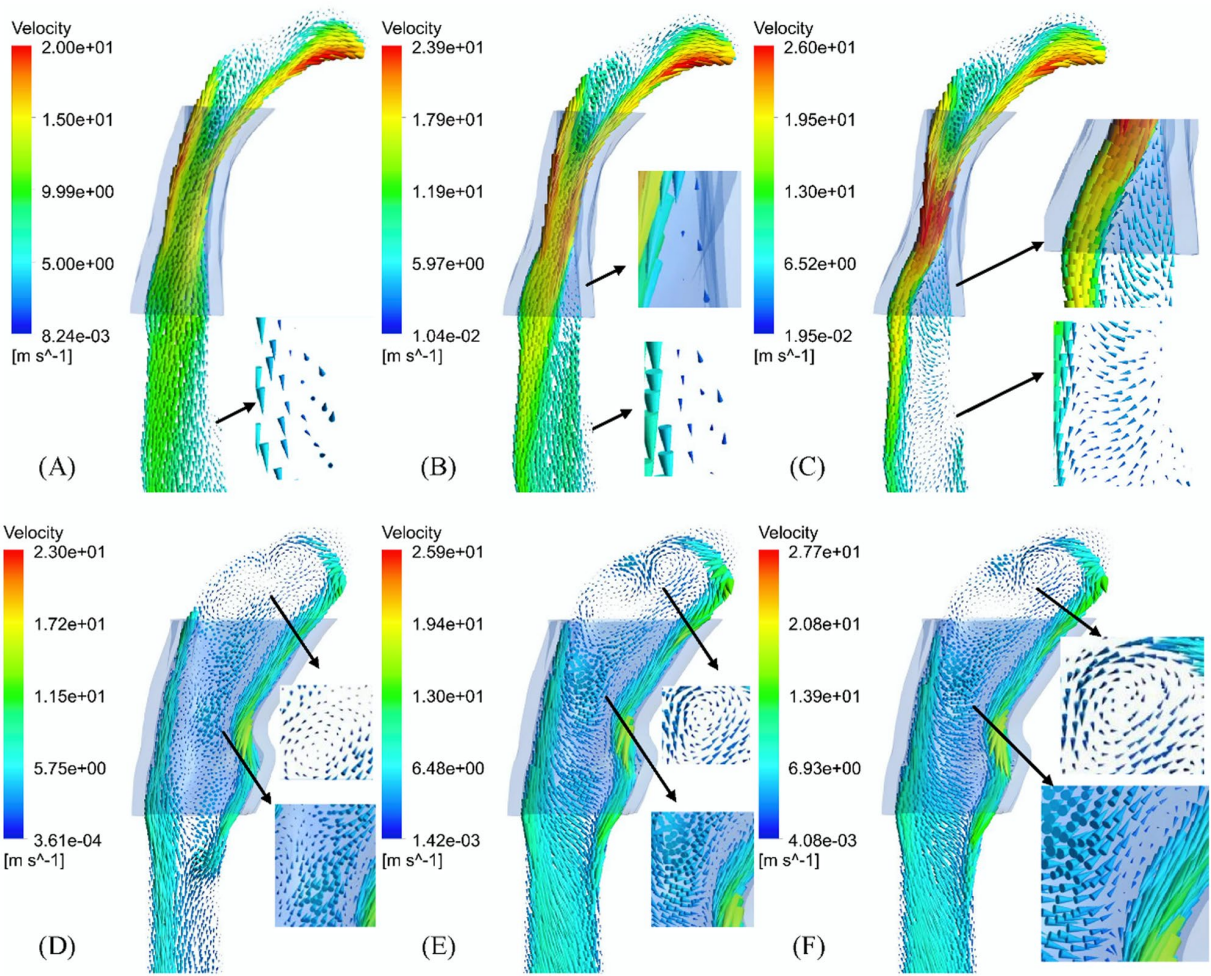
Velocity vector diagram of upper airway profile (transparent shadow is initial boundary of oropharynx): (**A**) 0.6 s, (**B**) 0.8 s and (**C**) 1.0 s before treatment; (**D**) 0.6 s, (**E**) 0.8 s and (**F**) 1.0 s after treatment

Figure 3 shows the velocity contour lines at three different moments during the inspiration process. With the increase of inspiratory flow rate, the cross section of the middle oropharynx decreased, and the jet was formed in the middle and lower oropharynx. At 1.0 s, flow separation caused by high-speed jet caused an obvious vortex in the downstream of the narrow part of the oropharynx. The main recirculation occurred below the most restricted area of the upper airway, and the flow separated from the hypopharynx returned to the oropharynx (Fig. 4). After Twin Block treatment, there was no obvious jet flow with the morphological changes in the oropharynx region, and the maximum velocity of upper airway decreased from 58.01 m/s before treatment to 46.16 m/s, and the maximum velocity of oropharynx decreased from 33.62 m/s before treatment to 28.26 m/s. However, two recirculating return zones consistently persisted in the same area of the nasopharynx and oropharynx during the inspiratory process.

#### Upper airway flow pressure

**Fig. 5:**
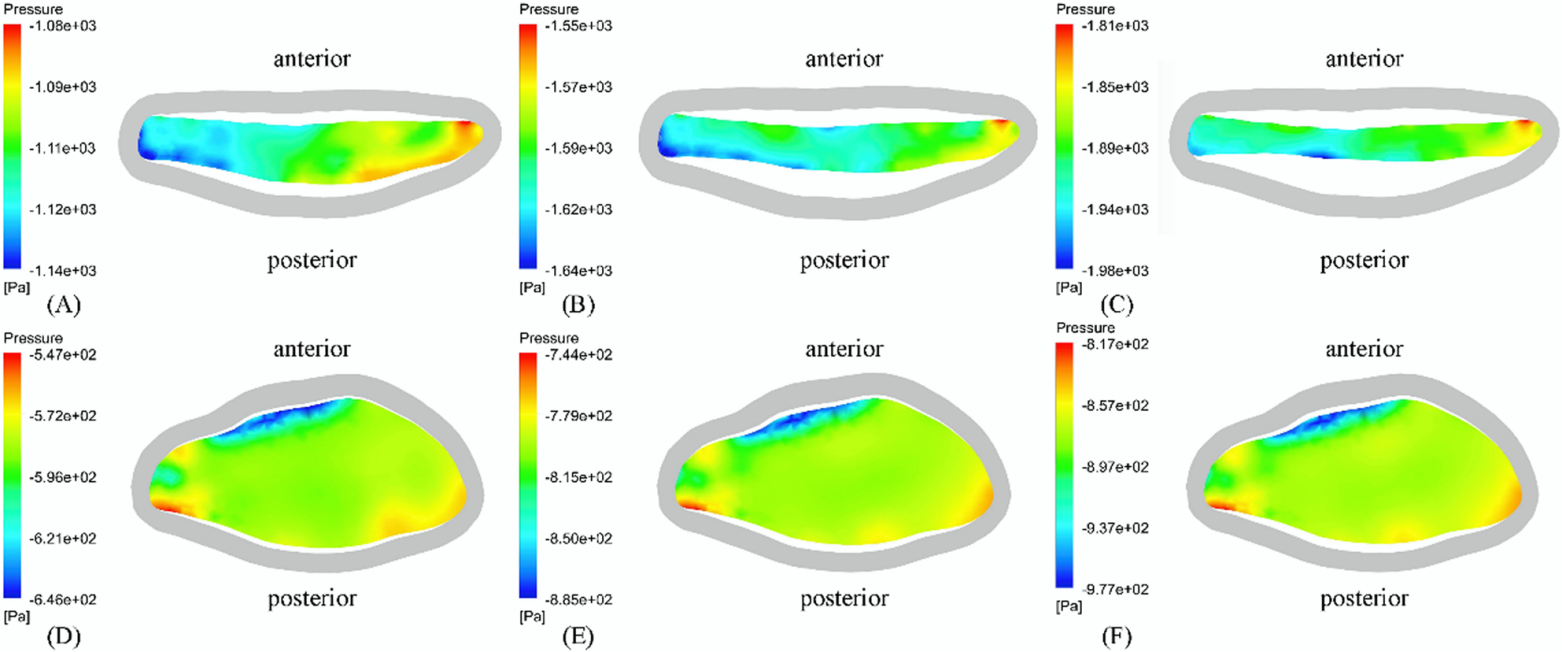
Pressure contour map of minimum cross-sectional area at peak inspiratory moment (gray shadow is initial boundary of oropharynx): (**A**) 0.6 s, (**B**) 0.8 s and (**C**) 1.0 s before treatment; (**D**) 0.6 s, (**E**) 0.8 s and (**F**) 1.0 s after treatment

**Fig. 6:**
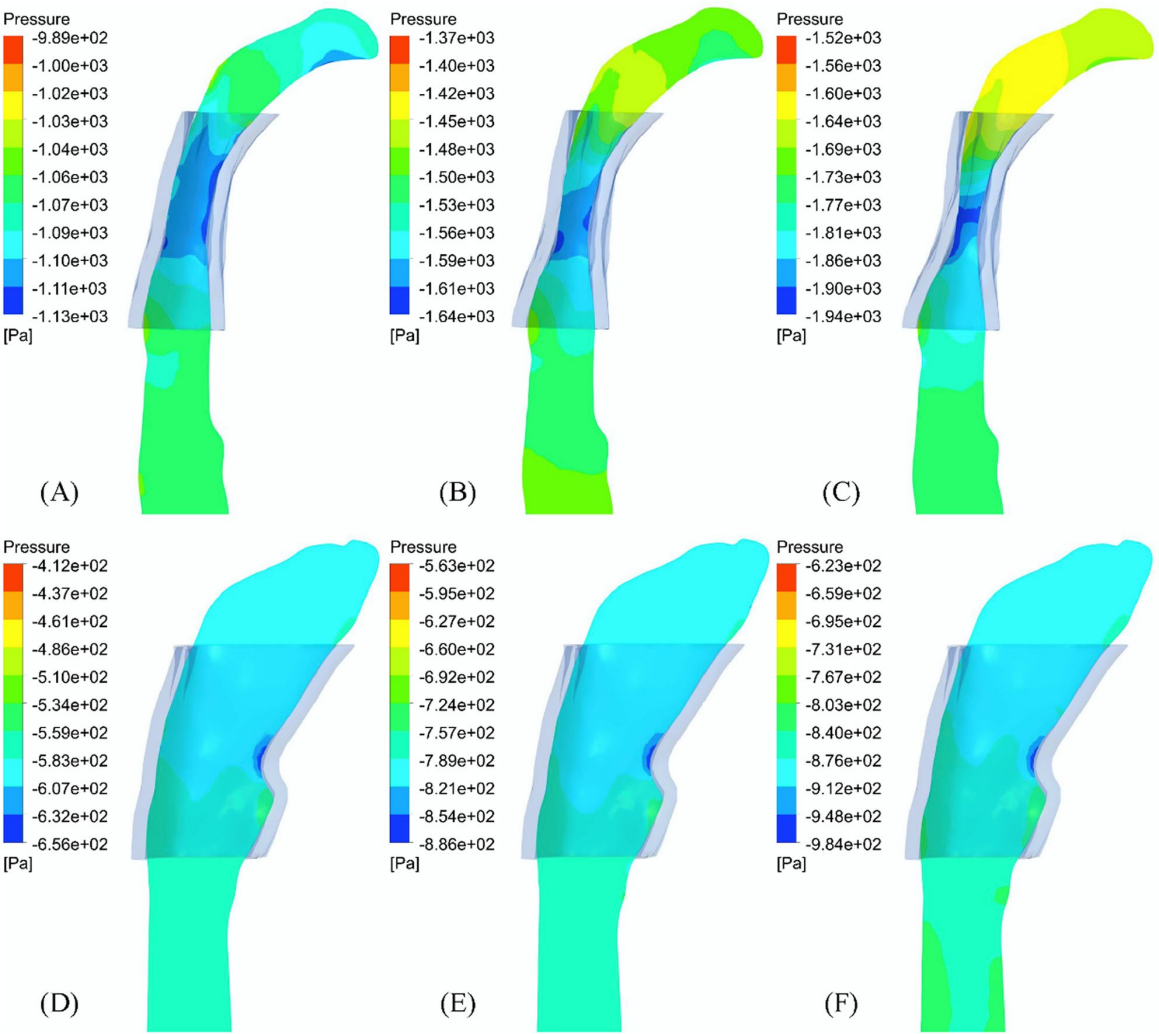
Pressure contour map of upper airway profile (transparent shadow is the initial boundary of oropharynx): (**A**) 0.6 s, (**B**) 0.8 s and (**C**) 1.0 s before treatment; (**D**) 0.6 s, (**E**) 0.8 s and (**F**) 1.0 s after treatment

The pressure contour map of the minimum cross-sectional area at the peak of inspiration before treatment revealed a larger pressure difference between the left-right direction compared to the anterior-posterior direction. However, due to the limitation that the significantly smaller diameter of the cross section in anterior-posterior direction (depth) compared to left-right direction (width), the oropharynx tended to be deformed mainly in the sagittal direction, which was consistent with the morphological characteristics of the cross section. There was a low pressure area in the posterior wall of the oropharynx, and since the FSI simulates only left-sided nasal inspirations, the negative pressure is mainly on the left side (Fig. 5). The relatively low pressure zone resulted from the presence of a “jet” in the oropharynx, resulting in a larger pressure gradient in the oropharynx than in the rest of the region (Fig. 6). With the passage of time, with the decrease of oropharynx pressure, the pressure difference between the internal oropharynx pressure near the anterior wall and posterior wall and the external pressure will increase, and this pressure difference will gradually make the anterior and posterior walls of the oropharynx closer and closer. After TB treatment, the maximum negative pressure was confined to a small area of the anterior oropharynx wall, which decreased from − 2741.81 Pa before treatment to −1767.54 Pa, and the pressure gradient also decreased significantly. The pressure difference in the left-right direction was smaller than that in the anterior-posterior direction, and the difference between the width and the depth was also smaller. The upper airway was also mainly deformed along the sagittal direction. In addition, the maximum resistance of upper airway decreased from 1.45 Pa/mL/s to 0.92 Pa/mL/s, and the maximum resistance of oropharynx decreased from 0.38 Pa/mL/s to 0.21 Pa/mL/s.

### Structural domain analysis

#### Oropharynx deformation and compliance

**Fig. 7:**
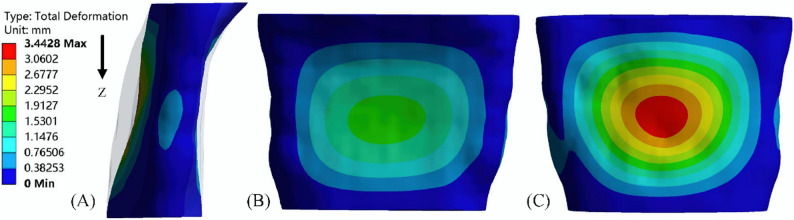
Oropharynx deformation 1.0 s before treatment (transparent shadow is the initial boundary of the oropharynx): (**A**) lateral oropharynx; (**B**) anterior wall of oropharynx; (**C**) posterior wall of oropharynx

**Fig. 8:**
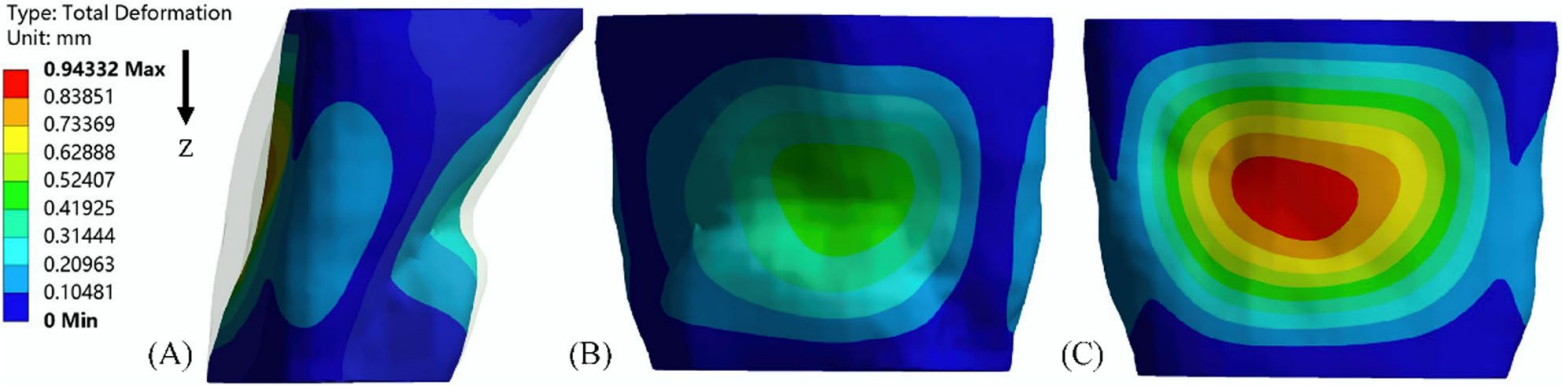
Oropharynx deformation 1.0 s after treatment (transparent shadow is the initial boundary of the oropharynx): (**A**) lateral oropharynx; (**B**) anterior wall of oropharynx; (**C**) posterior wall of oropharynx

**Fig. 9:**
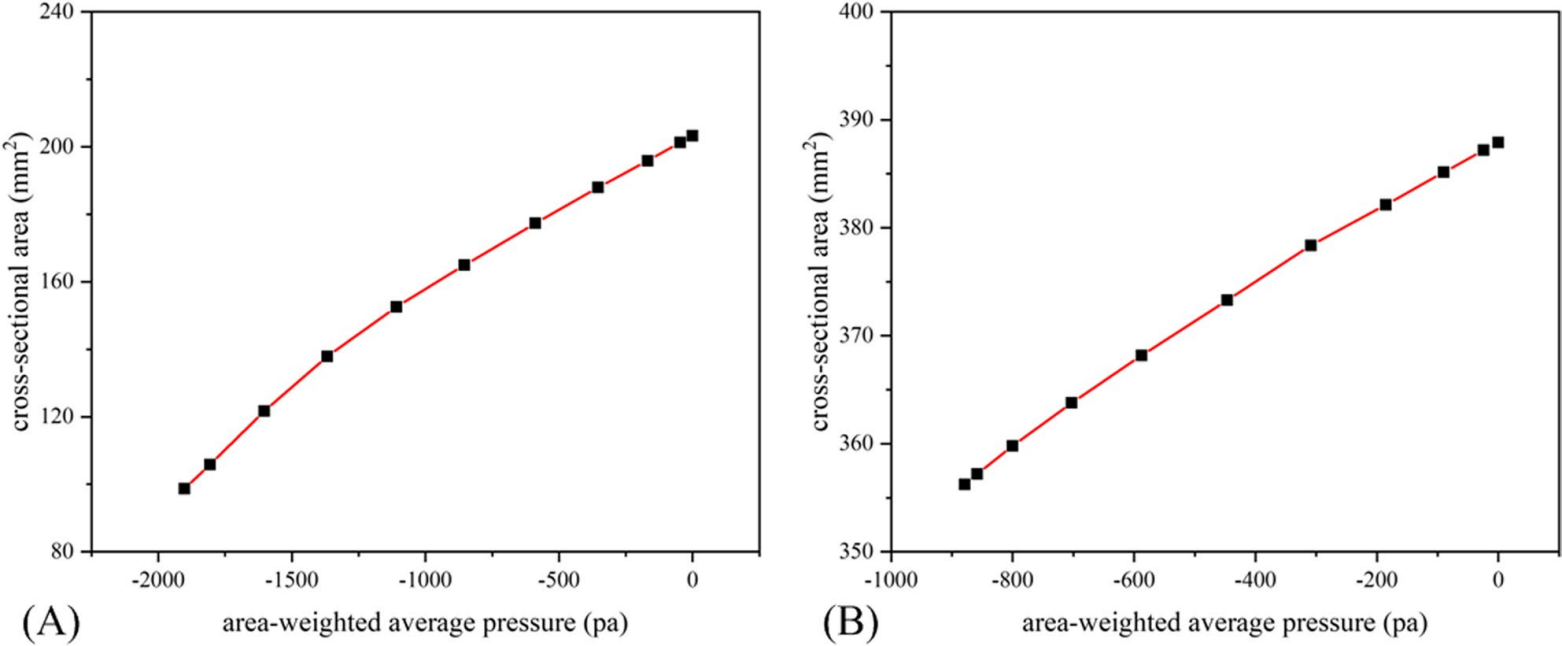
Area-pressure correlation curve: (**A**) before treatment; (**B**) after treatment

The deformation of the oropharynx before treatment is shown in Fig. 7, showing the position where the oropharynx begins to collapse. The chromaticity diagram represents the displacement of the deformation. The anterior and posterior walls of the oropharynx gradually moved closer to the center due to the pressure drop during inspiration. The largest deformation was located near the center of the posterior wall, reaching 3.44 mm. After treatment, the maximum deformation site remained in the posterior wall of the oropharynx, but the maximum deformation was reduced to less than 1 mm, which was reduced by more than 70%. (Fig. 8). Meanwhile, the compliance of the oropharynx soft tissue decreased from 0.048 mm^2^/Pa before treatment to 0.036 mm^2^/Pa after treatment (Fig. 9).

**Fig. 10:**
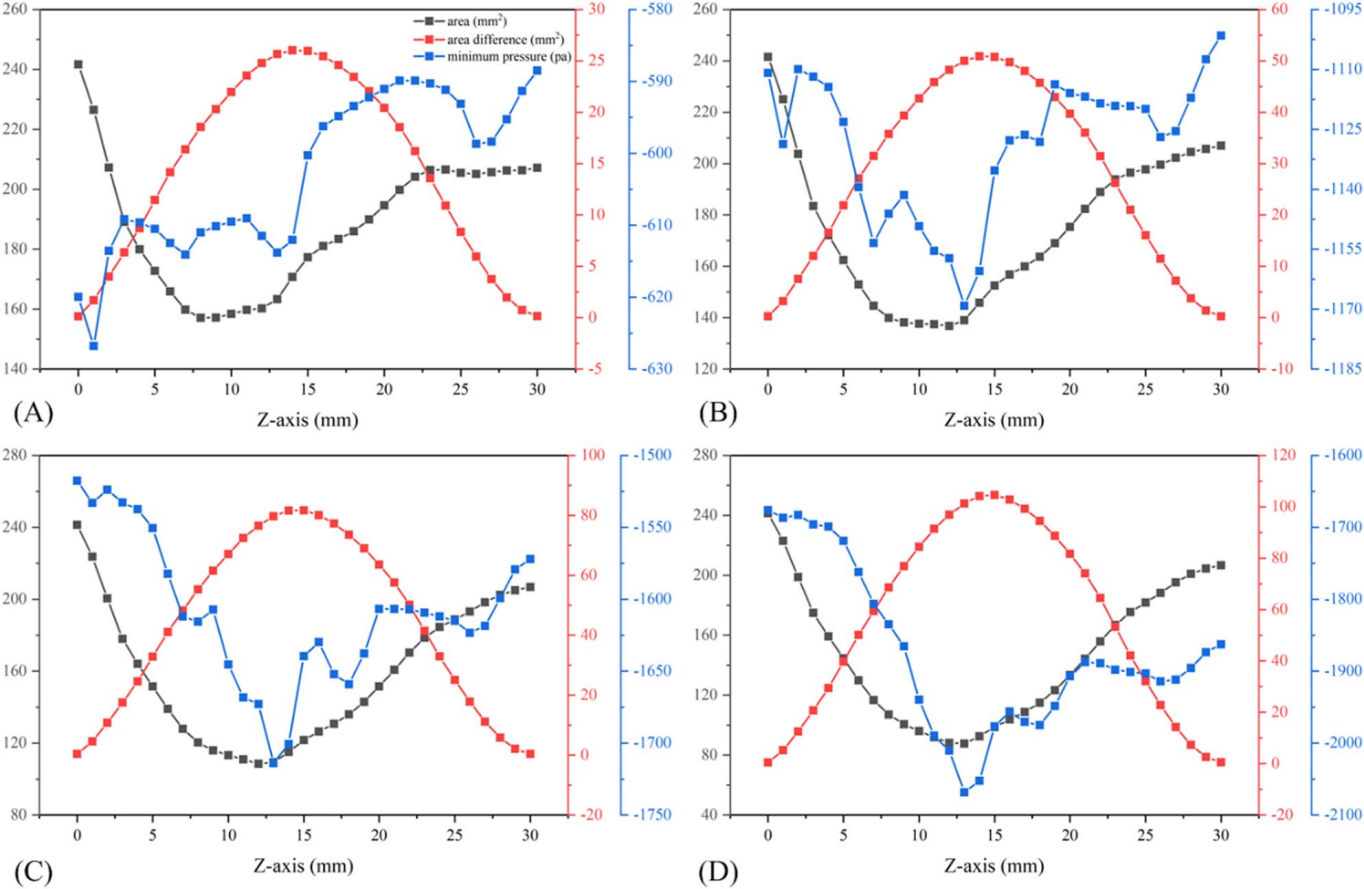
Changes of oropharynx cross-sectional area (black line), area difference (red line) and minimum pressure (blue line) along the Z-axis before treatment: (**A**) 0.4 s; (**B**) 0.6 s; (**C**) 0.8 s; (**D**) 1.0s

**Fig. 11:**
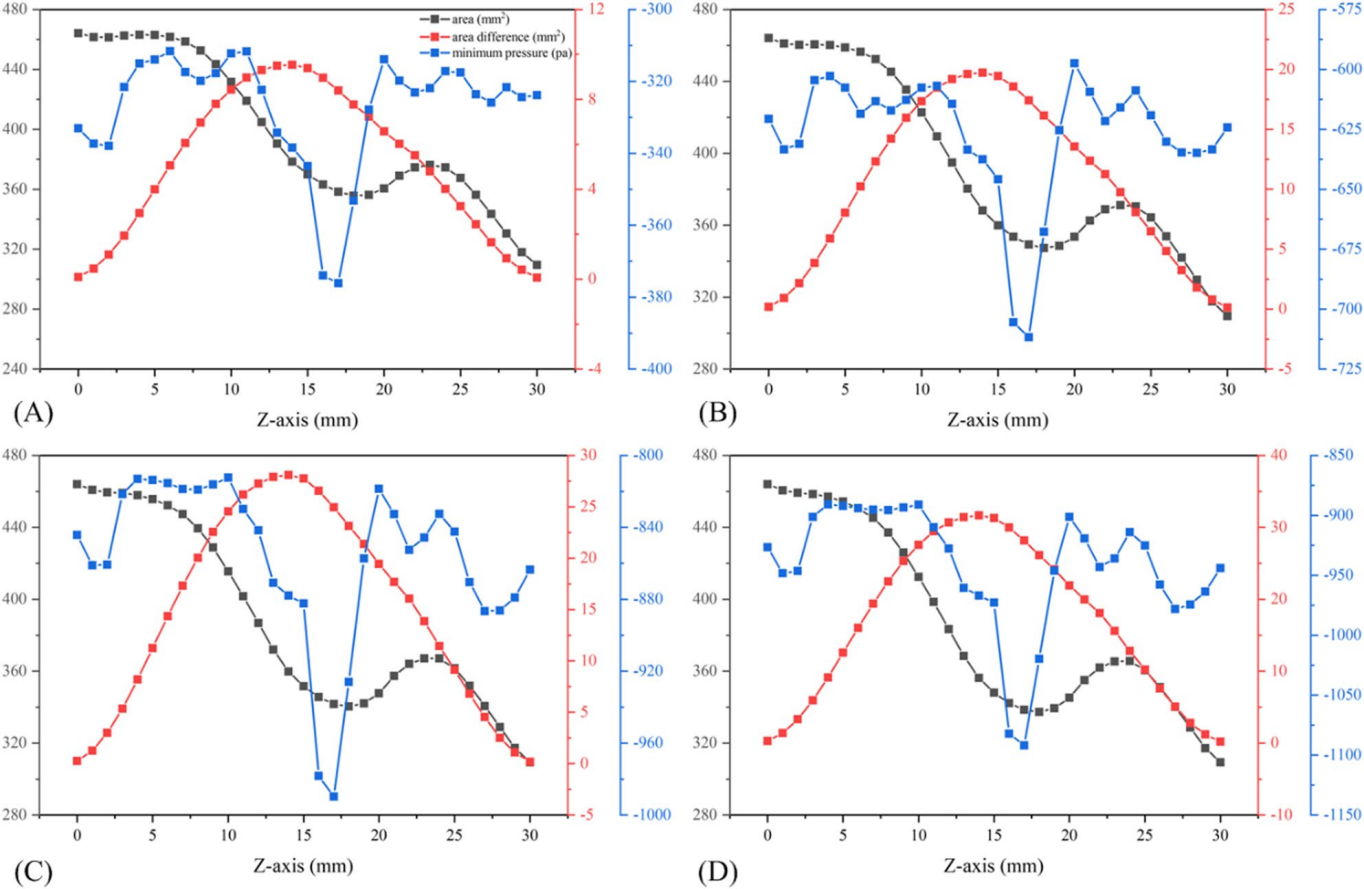
Changes of oropharynx cross-sectional area (black line), area difference (red line) and minimum pressure (blue line) along the Z-axis after treatment: (**A**) 0.4 s; (**B**) 0.6 s; (**C**) 0.8 s; (**D**) 1.0 s

**Fig. 12:**
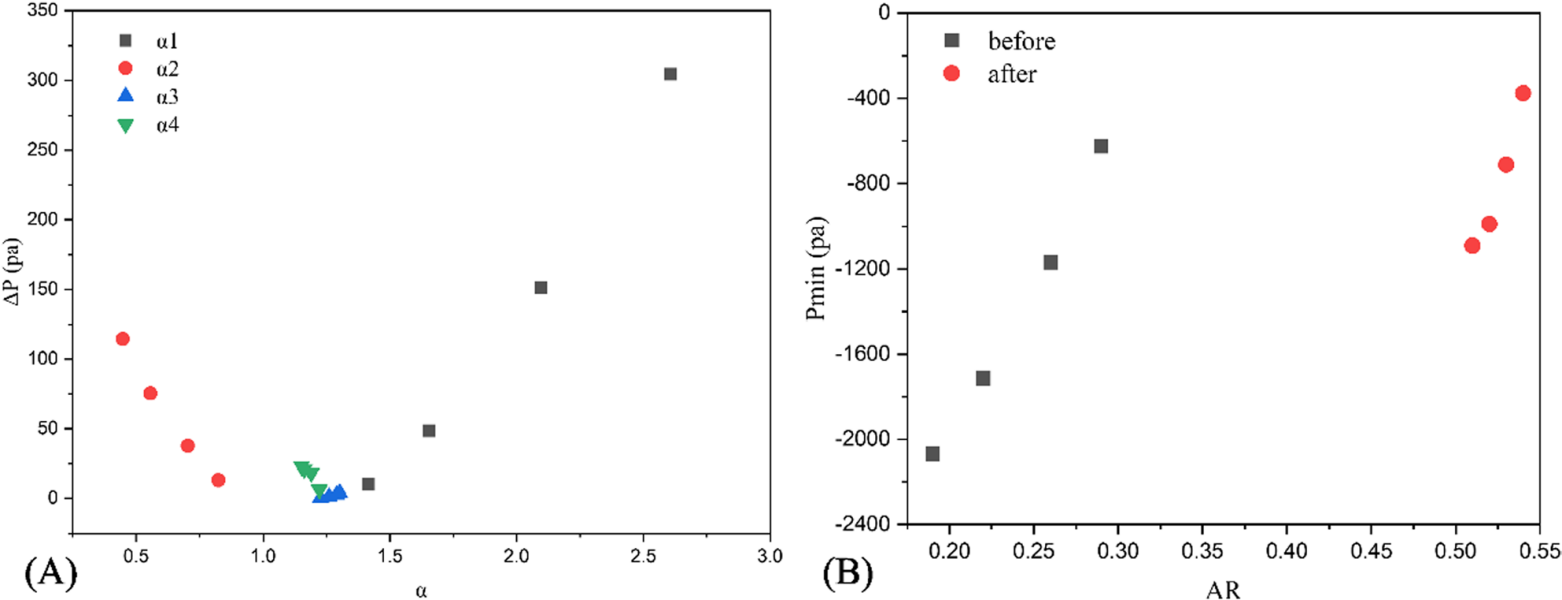
Hydrodynamic parameters of upper airway and relevant parameters of area morphology: (**A**) The correlation between pressure drop ΔP and cross-sectional area change rate α; (**B**) Correlation between minimum pressure Pmin and aspect ratio AR

Figure 10 shows the correlation between cross-sectional area, its changes, and pressure before treatment. The oropharynx soft tissues were divided into 30 cross sections along the Z-axis from top to bottom. The area of each cross section, the difference from the initial area, and the minimum pressure were calculated at the breathing time points of 0.4 s, 0.6 s, 0.8 s, and 1.0 s. The position at 8 mm was the position of the initial minimum cross-section before treatment, and the maximum negative pressure was always downstream during inspiration. For the minimum cross-sectional area at each breathing moment, the maximum negative pressure at the beginning of inspiration was also located downstream. However, as time progressed and the inspiratory flow rate increased, the two areas gradually converged. For each breathing moment, the area with the largest deformation was not at the initial minimum cross-section location, but near the minimum pressure, which also corresponded to the region with the largest longitudinal pressure difference relative to the upper and lower sections of the oropharynx. For each breathing moment after treatment, the area with the most significant reduction and the smallest pressure was not at the initial minimum cross-section (Fig. 11). Pearson correlation test showed that α was positively correlated with pressure drop (*r* = 0.687, *P* < 0.05), and the closer the α value approached 1, the smaller the oropharynx pressure drop. In addition, a larger AR associated with a smaller maximum negative pressure, and a significant negative correlation was observed between the two (*r* = 0.718, *P* < 0.05) (Fig. 12).

#### Prediction of oropharynx wall contact and collapse

**Fig. 13:**
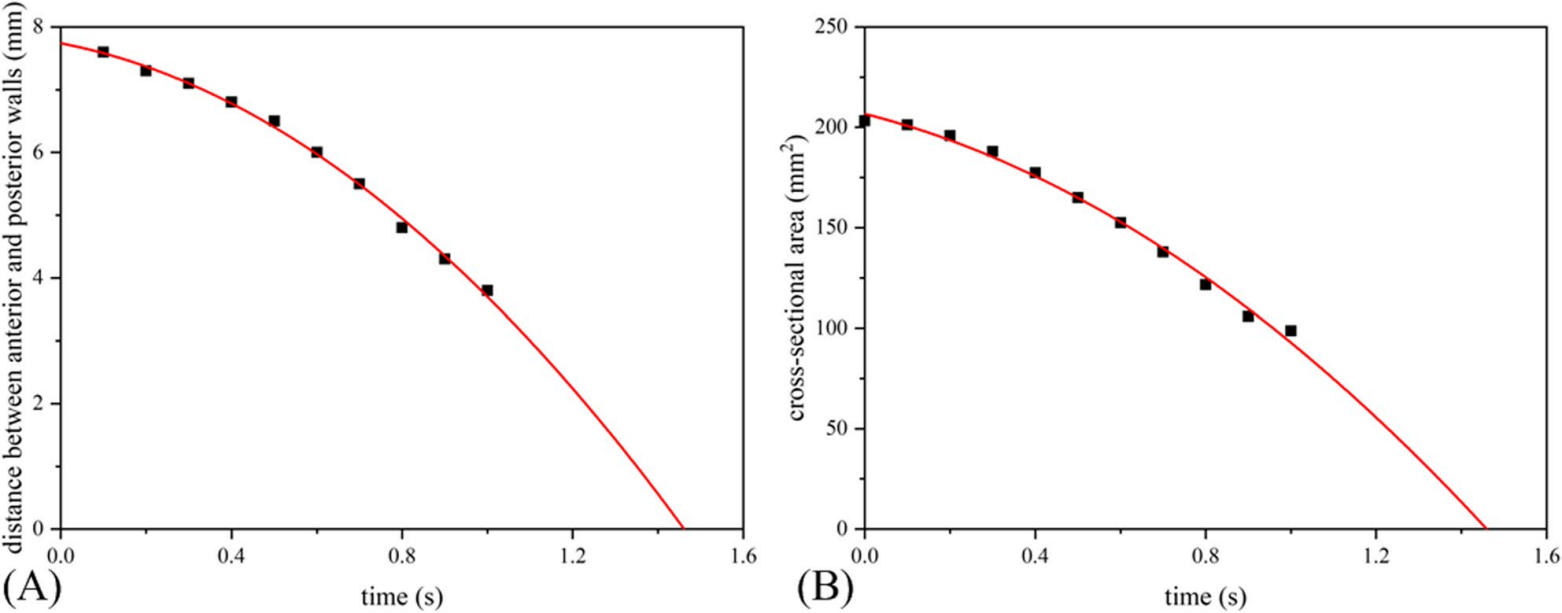
(**A**) Prediction curve of contact between anterior and posterior oropharynx walls; (**B**) Prediction curve of complete closure of the oropharynx soft tissue

Complete collapse of the upper airway cannot be reproduced by FSI alone. Therefore, based on polynomial curve fitting, the time of upper airway collapse can be predicted [26]. The results showed that the anterior oropharynx wall contacted the posterior wall at 1.45 s. At 1.47 s, the oropharynx soft tissue collapsed completely, and the upper airway cross-sectional area became zero (Fig. 13).

## Discussion

This study reconstructed a FSI model of the upper airway and oropharynx soft tissue based on the CBCT data of a patient, evaluated the interaction between the upper airway airflow and oropharynx soft tissue after TB, and validated the reliability of the method using a 3D printing model. Compared to the tissue thickness of OSAHS patients, the in vitro model used in this study has a smaller wall thickness to match the use of soft rubber materials that have less compliance than human pharyngeal tissue. The results showed that the anatomical structure of OSAHS collapse was not always the main location of flow restriction [27], and the contribution of each anatomical structure to flow restriction can be accurately quantified through computational fluid dynamics simulation [28].

During the inspiration process, the airflow flowing through the oropharynx from the nasal cavity mainly flowed downstream along the anterior and posterior walls of the upper airway, especially the anterior wall. Therefore, the maximum negative pressure was located in the anterior wall of the oropharynx. Zhao et al. also found that the pressure at the location where the airflow adheres was lower [9, 29], and this pressure distribution was highly correlated with the collapse mode of the upper airway [9, 29], resulting in a large displacement of the posterior wall forward. Consistent with the observation in vivo, as the inspiratory velocity increased, the oropharynx gradually narrowed, and the airflow further accelerated, forming the so-called oropharynx jet [14]. The downstream flow separation caused by it will cause energy loss [30], further reducing static pressure. The jet can also have a strong impact on the airway wall, causing high-frequency vibration of nearby soft tissues, leading to snoring and hypoventilation [31]. At the same time, a greater degree of narrowing of the oropharynx will lead to a further increase in the pressure difference between the inner and outer walls, which is the reason for the greater deformation of the oropharynx wall during inspiration, and also the reason for the difference in pressure predicted by CFD and FSI [14]. Thus, compared to CFD simulations that assume that the oropharynx wall is rigid, the negative pressure in FSI simulations will increase. It is generally believed in previous studies that the minimum cross-sectional area in the abnormal upper airway anatomy of OSAHS patients is the most relevant variable for the pathogenesis [32]. However, this study found that the location of the maximum deformation did not match the location of the initial minimum cross-sectional area, but was not far downstream of the narrowest part of the oropharynx, indicating that the site of OSAHS onset was not necessarily consistent with the narrowest part of the upper airway. After treatment, the positive effect of TB was confirmed by significantly increased pressure and small pharyngeal wall deformation. The maximum negative pressure was reduced by an order of magnitude, confined to a small area of the anterior oropharynx wall, and the maximum deformation was also reduced by 70%.

Polynomial curve fitting results showed that upper airway collapse occurred during inspiratory process, which is consistent with the in vivo observation that the airway narrowed during inspiratory period and widened during expiratory period [14], suggesting that the changes in respiratory function of inspiratory phase should be paid more attention in clinical treatment. On the other hand, from a mechanical perspective, the upper airway collapse depends on the contrast of forces that maintain its opening and promote its closure, which is mainly determined by transmural pressure and airway wall surface tension. Most scholars believed that the main force causing the closure of pharyngeal airway was the negative pressure in the airway, or the imbalance between the inward narrowing force of pharyngeal cavity and the outward expansion force generated by pharyngeal muscle. The upper airway compliance was an important factor in determining transmural pressure, and the shape of the airway section also played a crucial role in resisting deformation and collapse. In this study, the presented patient had mandibular retrognathia and exhibited an upper airway morphology that tended to have reduced anteroposterior depth and increased transversal width. FSI results showed that the oropharynx of such patients tended to have centripetal collapse in sagittal direction. Although the pressure difference in the left and right directions of the minimum cross section before and after treatment was larger than that in the sagittal direction, such pressure distribution did not significantly affect the collapse mode of the upper airway, and the maximum deformation was located near the maximum negative pressure of the anterior and posterior walls of the airway, which was consistent with the morphological characteristics of the cross section. The studies of Pirnar and Na et al. also showed that the parts of pharyngeal cavity with high negative static pressure were prone to collapse [14, 33]. Pearson correlation test showed that the relative size of the anteroposterior depth and transversal width was closely related to the maximum negative pressure of the upper airway. When the ratio difference between the two was too large, the oropharynx soft tissue was prone to deformation along the direction of smaller size. In addition, the contraction of the oropharynx caused significant changes in the fluid dynamics downstream of the airway. After TB, the anterior and posterior diameters increased significantly, the α value was closer to 1, the sectional shape was more symmetrical, and the change of pressure drop was smaller [25]. At the same time, pharyngeal compliance was also affected by airway geometry [10], and was reduced by 25%. Under the combined action of the above factors, the maximum negative pressure increased significantly and the maximum deformation decreased significantly after treatment. Although based on a single case, the consistent correlation between pressure gradients and deformation patterns before and after orthodontic treatment suggested this mechanism may generalize to other mandibular retrognathia cases.

There are still some limitations in this study. Firstly, the present FSI simulation of the upper airway and surrounding soft tissues has simplified the tissues around the upper airway. However, the biomechanical characteristics of the soft tissues around the upper airway are different, and the difference of its mechanical parameters may affect the changes of the fluid dynamics characteristics of the upper airway. Secondly, only one patient was included in this paper. The research results may not be representative and still need further verification. In future research, the sample size can be expanded and biomechanical parameters of the surrounding tissues of the upper airway can be measured for each individual in a specific state, establishing an individual specific upper airway biomechanical model, thereby providing reliable basis for the etiological diagnosis and treatment of each OSAHS patient.

## Conclusion

Based on FSI method, in this study, it was indicated that the minimum cross-sectional area of the upper airway did not match with the spatial position of the maximum deformation, and the variation in the cross-sectional shape of the upper airway and the distribution of the minimum pressure were closely related to the stability of the upper airway. This highlights the importance of combining soft tissue and airflow analysis to identify the collapse site and develop the optimal treatment plan. Moreover, the Twin Block appliance is helpful to enhance upper airway ventilation function in children with mandibular retrognathia.

## Data Availability

The datasets used and/or analyzed during the current study are available from the corresponding author on reasonable request.

## Abbreviations

CFD: Computational fluid dynamics
FSI: Fluid-structure interaction
OSAHS: Obstructive sleep apnea-hypopnea syndrome
TB: Twin Block
CBCT: Cone-beam computed tomography
Mimics: Materialse's interactive medical image control system
STL: Stereolithography
AR: Aspect ratio
